# Is hysterosalpingo-foam sonography (HyFoSy) more tolerable in terms of pain and anxiety than hysterosalpingography (HSG)? A prospective real-world setting multicentre study

**DOI:** 10.1186/s12905-022-01606-3

**Published:** 2022-02-13

**Authors:** Lucía Serrano González, Tirso Pérez-Medina, Beatriz Bueno Olalla, Ana Royuela, María de los Reyes De La Cuesta, David Saéz de la Mata, Esther Domínguez-Franjo, Laura Calles-Sastre, Virginia Engels

**Affiliations:** 1 Department of Gynaecology and Obstetrics, Puerta de Hierro Majadahonda University Hospital, Joaquín Rodrigo St, 1, 28222 Majadahonda, Madrid Spain; 2 Department of Gynaecology and Obstetrics, Infanta Sofía University Hospital, Paseo de Europa, 34, 28703 San Sebastián de los Reyes, Madrid Spain; 3 Biostatistics Unit, Biomedical Research Institute Puerta de Hierro-Segovia de Arana (CIBERESP) ES, Joaquín Rodrigo St, 1, 28222 Majadahonda, Madrid Spain; 4 Department of Radio Diagnosis, Infanta Sofía University Hospital, Paseo de Europa, 34, 28703 San Sebastián de los Reyes, Madrid Spain; 5 Present Address: Department of Gynaecology and Obstetrics, Hospital Universitario Ramón y Cajal, M-607, km. 9, 100, 28034 Madrid, Madrid Spain

**Keywords:** Hysterosalpingo-foam sonography (HyFoSy), Hysterosalpingography (HSG), Tolerability, Pain, Anxiety

## Abstract

**Background:**

In 60% of sterile couples a female factor is present, with these being tubal factors in 30–50% of cases. A tubal patency test is also required in women without a male partner undergoing fertility treatment. Thus, an accurate, safe and tolerable technique should be available. The aim of this study is to determine and to compare hysterosalpingo-foam sonography (HyFoSy) and hysterosalpingography (HSG) tolerability in terms of pain and anxiety.

**Methods:**

This is a prospective real-world setting multicentre study conducted in two tertiary hospitals in Madrid. 210 infertile women/women without a male partner looking to get pregnant were recruited; 111 for the HyFoSy group and 99 for the HSG group. Tolerability was measured in terms of anxiety by the State Trait Anxiety Inventory (STAI) and pain by the Visual Analogue Scale (VAS).

**Results:**

Median VAS score in HyFoSy group was 2 (P25; P75: 1; 3) versus 5 (4; 8) in HSG group, *p* < 0.001. The median State-STAI score in the HSG group was 18 points (10; 26) versus 10 (7; 16) in the HyFoSy group (*p* < 0.001); the median Trait-STAI score in the HSG group was 15 (11; 21) versus 13 (9; 17) in the HyFoSy group (*p* = 0.044).

**Conclusions:**

HyFoSy shows higher tolerability to both: pain and anxiety. It is related to less pain and less post-test anxiety than HSG.

## Background

Our society faces an increasing prevalence of fertility problems (estimated 10–20% of couples); this increase is mainly associated with the delay in trying for a first child [1]. Approximately sixty per cent of cases are related to female sterility, including those cases with female factor and those with female and male factor [1, 2], with 30–50% of them being due to tubal factors [3–5]. Thus, tubal patency should be assessed in many infertile couples. The tubal patency test is also necessary for women without a male partner who want to get pregnant, prior to indicating intrauterine insemination with donor sperm. In all cases, it is important to offer a reliable, safe and tolerable technique. The gold standard for assessing tubal patency is laparoscopy and dye chromopertubation (lap-and-dye test). However, this technique is rarely used because it is both invasive and expensive. Therefore, HSG is the technique that is largely employed in most countries to date. This approach is a precise, safe, effective and economical technique. Nevertheless, HSG also has clear disadvantages that force us to look for an alternative: it is painful, employs iodinated contrast and exposes the patient to ionising radiation.

Hysterosalpingo-contrast sonography (HyCoSy) is a newer sonographic technique, which uses ultrasounds (avoiding ionising radiation) and non-iodinated contrast to assess the uterine cavity and fallopian tubes; when the contrast employed is a foam, like in our study, it can be called hysetosalpingo-foam sonography (HyFoSy). There is consistent evidence that supports its reliability. It has shown a high detection rate of tubal obstruction and good reproducibility [3, 6–8], with concordances from 83.8 [9] to 100% [10] with HSG, and from 78.1 [11] to 96.91% [12] with lap-and-dye test. In addition, this technique permits an accurate evaluation of uterine cavity, with concordance with hysteroscopy up to 100% [13, 14]. HyCoSy/HyFoSy has also been shown to be a safe technique [15–18]. In addition, it allows for a single comprehensive assessment of the uterus and the junctional area and it can be performed by the same specialist who indicates it, in the clinician’s own office [19], with it consequently being time efficient. Finally, some studies have shown an increase in the rate of post-procedure gestation [20–23]; nevertheless, to confirm this effect, well established for HSG, more studies are needed. It has been suggested in the literature that HyCoSy/HyFoSy replace HSG for tubal assessment [3, 7, 10, 11, 14, 15, 17, 18, 24–27].

The aim of this study was to assess the tolerability of HyFoSy in terms of pain and anxiety and to compare it with the tolerability of HSG, in order to obtain evidence that would allow for a change in first-line tubal patency test. We chose to perform a real-world setting study because a different diagnosis procedure for tubal patency testing is protocolised in each centre: HyFoSy at Puerta de Hierro Majadahonda University Hospital and HSG at Infanta Sofia University Hospital. Real-world studies have become increasingly important in providing evidence; one of the main features thereof is the use of data collected outside the narrow confines of conventional randomised controlled trials to evaluate what is happening in normal clinical practice, reflecting usual care [28].

## Methods

This prospective multicentre study was conducted with the consecutive data of 210 women from the Reproduction Unit at Puerta de Hierro Majadahonda University Hospital and Infanta Sofia University Hospital between September 2017 and October 2018, the time needed to ensure a sufficient sample size, calculated in line with VAS values for both techniques found in the literature [15, 17, 29–32], and with the aim of being both conservative and achieving a sufficient sample size to ensure adequate power to identify differences in tolerability hypothesis contrast in terms of anxiety. For the calculation thereof, we did not have reference values from the literature. Both centres belong to Public Health Service of Madrid and attend to similar populations. Women included in this study were seen at their referral hospital and thus either underwent HSG (Infanta Sofía University Hospital) or HyFoSy (Puerta de Hierro Majadahonda University Hospital).

Ethical approval was granted by the Institutional Review Board of Puerta de Hierro Majadahonda University Hospital (Number 12.17; Majadahonda, Madrid, Spain) and by the Institutional Review Board of Infanta Sofia University Hospital (number 5.7.17; San Sebastián de los Reyes, Madrid, Spain) prior to starting data collection. All the patients signed an informed consent before being included in the study. The inclusion criteria were women aged 18–40 years undergoing assisted reproduction that required a tubal patency test, including women with a male partner and a history of sterility/infertility (have not achieved ongoing pregnancy leading to live birth after at least a year of search) [33], women with a female partner and single women who wished to become pregnant. The exclusion criteria were current pregnancy, heavy menstrual bleeding, tubal patency previously assessed, psychiatric pathology, treatment with psychotropic drugs, severe male factor (R motile sperm count ≤ 3 × 10^6^), allergy to contrast media or to premedication drugs and pelvic inflammatory disease.

Thus, the patients were recruited and scheduled to undergo a tubal patency test as follows: HSG at Infanta Sofia University Hospital (the hospital’s routine procedure since opening in April 2007), and HyFoSy at Puerta de Hierro Majadahonda University Hospital (the hospital’s routine procedure since September 2015); as this was a real-world setting study, the routine procedure at each centre was not altered and was performed as is standard.

The main outcomes of our study were pain and anxiety. In order to quantify pain, we employed the Visual Analogue Scale (VAS) scored in centimetres from 0 (no pain) to 10 (the worst imaginable pain) [34]. In order to assess anxiety we employed the State Trait Anxiety Inventory (STAI) questionnaire; this validated instrument for the measurement of anxiety consists of two scales: Trait-STAI, to measure basal anxiety, and State-STAI, to measure anxiety at a given moment; on both scales, the higher score, the higher the anxiety (from 0 to 60 points) [35].

When patients were recruited they filled out a Trait-STAI questionnaire. The patency test was scheduled in the immediate postmenstrual phase (days 6–12). Patients were premedicated with oral azithromycin (1 g) the night before, and with a step 1 WHO Pain Ladder drug [36] 1 h before the test. Just after the test, patients were asked to report pain intensity experienced using VAS and to fill out a State-STAI questionnaire. The patients remained under observation for 30 min to identify and treat any immediate side effects and complications that may have occurred after the test.

HSGs were carried out at the Radio Diagnosis Department of Infanta Sofia University Hospital. The professionals involved included a nurse trained to perform the technique, who performed cervical cannulation and contrast instillation, an X-ray technician who assisted both patient and nurse and captured and processed images, and a radiologist who interpreted the images and wrote a deferred report. Cervical cannulation was performed with the patient placed in the lithotomy position; the cervix was visualised with the aid of a disposable speculum (Bexen Medical®). Then, the cervix was cannulated using a HSG disposable set with 5Fr catheter with anti-reflux balloon (REDI-TECH®, Atlanta, GA). The anti-reflux balloon was placed in the cervical canal and filled with 2 ml of sterile water. The difficulty of cervical cannulation was classified as low difficulty for a centred cervix and smooth entrance, medium difficulty for a lateralised cervix and/or some degree of hampering, and high difficulty for those cases that required cervical traction with a Pozzi clamp to straighten the cervical angle. Once the catheter was positioned, Visipaque TM 270 mg/ml (GE HealthCare®), water-soluble iodinated contrast media, was instilled in bolus under fluoroscopy until passage of the contrast to the peritoneal cavity was seen or until the passage offered resistance (10 ml maximum). Tubal patency was determined by the passage of the contrast media into the peritoneal cavity within two minutes. A C-shaped fluoroscopy arc (Siemens®) was used to capture the images, allowing a fluoroscopy with removal of bone tissue to visualise the uterus and the passage/blockage of the contrast through the tubes. Then, the speculum was removed. Representative photographs were taken in left and right oblique projection and in antero-posterior projection. Finally, the anti-reflux balloon was deflated and the catheter was removed. The test report was given to the patient in a subsequent appointment at the Reproduction Unit.

HyFoSy was performed by two gynaecologists: one of them inserted the intracervical catheter and instilled the contrast and the other performed the ultrasound. A nurse prepared the contrast media and assisted both patient and gynaecologists. Ultrasound was performed with a GE Voluson 730 Pro ultrasound device (GE Healthcare, Milwaukee, WI, USA) equipped with 3D-transvaginal probe (6–12 MHz) and 2D-abdominal probe (2–7 MHz). We employed ExEm Foam® as contrast. It contains hydroxyethyl cellulose and glycerol dissolved in purified water just before the procedure to form foam that enables very clear ultrasonography visualisation of the uterine cavity and the tubes. For insertion of the intracervical catheter, the patients were placed in the lithotomy position. After the cervix was visualised with a disposable speculum (Unidix®), a paediatric nasogastric tube (Unomedical CH6, ConvaTec Ltd, Deeside, UK) was inserted with the help of a Foerster clamp (low difficulty). If cervix cannulation was not possible, a rigid insemination cannula (Kitazato® Hard—Long Type 6Fr) (medium difficulty) or even a Pozzi clamp gripping the cervix to straighten the cervical angle (high difficulty) were employed. Anti-reflux system was not used. The correct positioning of the catheter was verified by abdominal ultrasound and then the speculum was removed. Thereafter, an transvaginal ultrasound was performed in order to evaluate associated gynaecological disease and carry out a follicle count. The next step was to infuse 3 millilitre (ml) of ExEm Foam® by pushing the plunger of the syringe with light pressure. After identifying the contrast in the uterine cavity in the longitudinal plane, the gynaecologists first performed an automatic volume acquisition to enable 3D reconstruction of the uterine cavity in the coronal plane; the volumes were saved and evaluated offline immediately after the examination. Then, the transvaginal sonographic probe was rotated to the transverse plane to proceed with the evaluation of the fallopian tubes. If they were not visible seven minutes after contrast instillation, more contrast was instilled in small 1–2 ml boluses, up to 10 ml. If the tubes were not visible, they were considered not permeable. A video and photographs of the flow (or absence of flow) of the contrast through the tubes were taken. The test report was provided to the patient at the same visit.

Primary endpoints were pain intensity (VAS) and trait and state anxiety (STAI). Secondary endpoints were success/failure of the test, cervical cannulation difficulty, contrast media volume (ml) and tubal patency. The following patient characteristics were also collected: age, couple type (male partner, female partner, no partner) and medical and surgical history.

A descriptive analysis of the categorical variables was performed using absolute and relative frequencies and of the numerical variables, by means and standard deviation or median and percentiles 25 and 75 (P25; P75), according to compliance with the assumption of normality. Univariate analysis was performed using the Mann–Whitney U test to contrast numerical variables and Fisher’s exact or Chi-square tests for hypothesis tests of categorical variables, as appropriate. The level of significance was set at 0.05. The statistical package used was Stata/IC v.16 (StataCorp. 2019. Stata Statistical Software: Release 16. College Station, TX: StataCorp LLC.).

## Results

**Fig. 1:**
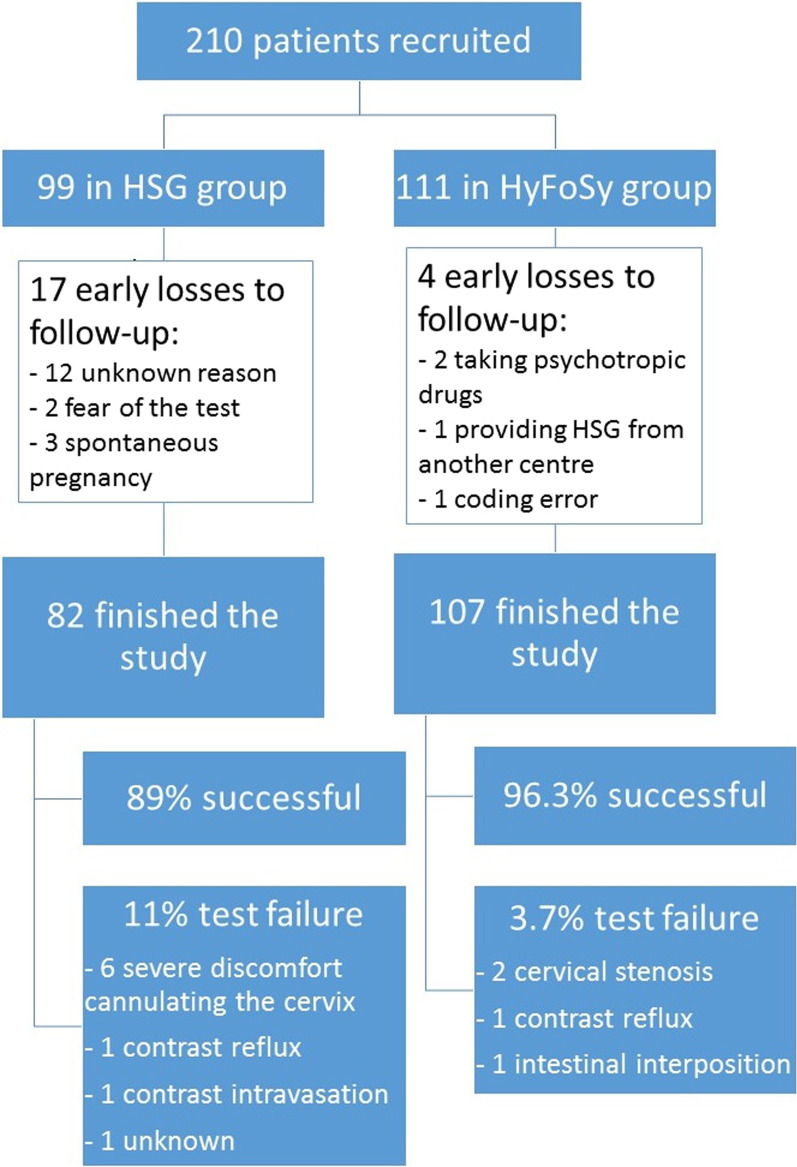
Flowchart of study participants. Patients recruited losses to follow-up and test failures

In the HSG group, 99 patients were recruited with 17 losses to follow-up before the procedure was performed. In the HyFoSy group, 111 patients were recruited, with 4 losses to follow-up before the procedure was performed (Fig. 1). The epidemiological characteristics of the patients are collected in Table 1. The tubal patency test was completed in 89% (73/82) of HSG patients and in 96.3% (103/107) of HyFoSy patients (Fig. 1). Failure in the HSG group is mainly related to tolerance factors (66.7% of test failures, 6/9); while in the HyFoSy group, the main cause was cervical stenosis (50% of test failures, 2/4) (Fig. 1).

**Table 1:** Population characteristics of patients

Characteristics	HSG group	HyFoSy Group	*p* value
Age	32.06 years (SD 3.1) (range 24–37)	34.63 years (SD 3.2) (range 25–40)	< 0.001
Couple type			
Male partner	91.5% (75/82)	90.7% (97/107)	0.975
Female partner	4.9% (4/82)	5.6% (6/107)	
No partner	3.7% (3/82)	3.7% (4/107)	
Medical history related to the internal genital tract	0% (0/82)	8.41% (9/107) (4) Endometriosis (1) Chorioamnionitis (1) Puerperal parametritis (1) Ulcerative colitis (1) Repeated abortions (1) Amenorrhea after uterine curettage	0.006
Abdominopelvic surgery	26.83% (22/82) (2 patients had undergone abdominopelvic surgery twice)	36.45% (39/107) (3 patients had undergone abdominopelvic surgery twice and 1 patient had undergone abdominopelvic surgery three times)	0.161
Uterine surgery	14.6% (12/82)	18.7% (20/107)	
Conisation	0% (0/82)	0% (0/107)	
Digestive system surgery	7.3% (6/82)	11.2% (12/107)	
Adnexal surgery	4.9% (4/82)	8.4% (9/107)	
Abdominal wall surgery	1.2% (1/82)	1.9% (2/107)	
Urologic surgery	1.2% (1/82)	0.9% (1/107)	

Cervical cannulation was classified as low difficulty in most patients in both groups (HSG group 65.8%, 48/73, HyFoSy group 89.3%, 92/103) and high difficulty was observed in 6.9% (5/73) of HSG patients versus 1.9% (2/103) of HyFoSy patients, being statistically significant (*p* = 0.001).

Contrast media volume instilled was 8.8 ± 4.1 ml/patient in the HSG group and 4.5 ± 2 ml/patient in the HyFoSy group (*p* < 0.001).

Regarding the results for tubal patency, they were similar in both groups (*p* = 0.267); bilateral obstruction ratio 3.7% (3/82) in the HSG group and 7.5% (8/107) in the HyFoSy group.

The median intensity of pain was 5 (4; 8) in the HSG group and 2 (1; 3) in the HyFoSy group (*p* < 0.001). The odds ratio (OR) for severe pain (VAS score ≥ 7) in the HSG group was 16.5 (95% CI 4.8; 57.1).

**Fig. 2:**
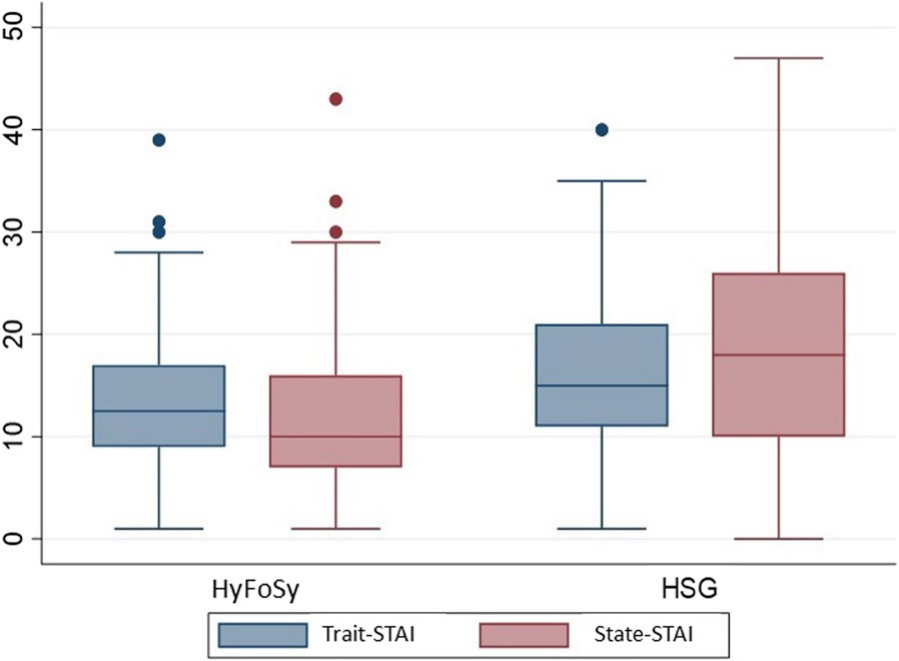
Trait-anxiety and state-anxiety. Trait-STAI questionnaire (how one generally feels) was filled out at the recruitment visit, while the State-STAI questionnaire (how one feels at the moment) was filled out just after the test. We can see how in the HSG group, anxiety increases following the procedure, while in the HyFoSy group it decreases. The median Trait-STAI score in the HSG group was 15 (11; 21) versus 13 (9; 17) in the HyFoSy group (*p* = 0.044); the median State-STAI score in the HSG group was 18 points (10; 26) versus 10 (7; 16) in the HyFoSy group (*p* < 0.001)

Concerning basal anxiety, the median Trait-STAI score in the HSG group was 15 (11; 21) versus 13 (9; 17) in the HyFoSy group (*p* = 0.044). As regards post-procedural anxiety, the median State-STAI score rose to 18 (10; 26) in the HSG group but dropped to 10 (7; 16) in the HyFoSy group (*p* < 0.001) (Fig. 2).

## Discussion

The main finding of the present study is the observation of greater tolerability to HyFoSy when compared to HSG, as it is less painful and causes less anxiety.

In this sense, there was a difference in the failure rate between HSG (11%, 9/82) and HyFoSy (3.7%, 4/107) (Fig. 1); a remarkable proportion of failures in the HSG group was due to tolerance factors (severe discomfort) (66.7%, 6/9).

A greater number of losses to follow-up were observed in the HSG group: 17.2% (17/99) versus 3.6% (4/111) in HyFoSy group (Fig. 1). The main cause of loss to follow-up in the HSG group was not attending the appointment for unknown reasons (70.6%, 12/17). Among appointment cancellations for known reasons, there were two main reasons: spontaneous gestation and fear of undergoing the test. Other authors reported HSG as the most feared test in the infertility process [37]. Seeking medical information online, especially gynaecological information, is increasingly common [38, 39] especially among young people [37] represented in the age rank of our study, even though the quality of this information is not guaranteed [39]. Handelzalts et al. [40] reported in 2010 that 79% of women who underwent HSG had sought information regarding the technique, mostly through the internet (91.4%). They found that in those who sought information anxiety was significantly higher (State-STAI score 16.8 vs. 13.2, *p* 0.016). Perhaps those patients who were scheduled to undergo HSG, who did not attend their appointment due to unknown reasons, were simply afraid of having the test and decided not to go through with it due to the negative opinions about HSG on Internet forums, social networks and websites.

Concerning tolerability in terms of anxiety, we found very few data available in the literature. Previous studies reported HSG as a highly stressful procedure [37, 40], but there are no previous studies that compare HyCoSy/HyFoSy and HSG in terms of anxiety induced by the procedure. Our results support the hypothesis that HyFoSy causes less anxiety than HSG. State anxiety (post-procedural anxiety) was significantly higher in HSG (median State-STAI score in the HSG group was 18 (10; 26) versus 10 (7; 16) in the HyFoSy group; *p* < 0.001), while trait anxiety (basal anxiety) did not differ greatly between groups (the median Trait-STAI score in the HSG group was 15 (11; 21) versus 13 (9; 17) in the HyFoSy group; *p* = 0.044). The observation of a similar trait anxiety score in both groups suggests that the difference in state anxiety reflects the techniques’ varying capacities to evoke anxiety.

Our explanation is that anxiety increases immediately after HSG and not after HyFoSy mainly because patients who undergo HSG experience more pain. On the other hand, it is striking that in the HyFoSy group anxiety decreases immediately after the test (median Trait-STAI score 13 (9; 17) vs. median State-STAI score 10 (7; 16)). We think that this could be because these patients receive the test report immediately from the gynaecologist who is dealing with their fertility problem; therefore, at that moment, any doubts they may have about the reproductive prognosis and treatments that may be required are resolved.

We ruled out the use of benzodiazepine as premedication because it is not supported by the literature. Nevertheless, we consider it important to implement measures to reduce anxiety in tubal assessment tests (counselling intervention, calm environment), as fear and anxiety increase discomfort during the procedure and are likely to influence perceived pain [29, 41, 42].

Concerning pain intensity, we found that HyFoSy is significantly less painful than HSG, with a median VAS score of 2 (1; 3) versus 5 (4; 8) (*p* < 0.001), using a step 1 World Health Organization Pain Ladder drug before the procedure. The OR for severe pain (VAS score ≥ 7) in the HSG group was 16.5 (95% CI 4.8; 57.1). A similar VAS score is reported by other authors for HSG [29, 30, 42], HyCoSy [15, 31] and HyFoSy [17, 31].

There are several reasons that explain the differences in the VAS score for these techniques. The first lies in cervical cannulation; in HSG it represents a very painful step and implies a high failure rate (7.31%, 6/82); a possible explanation for this could be that in HyFoSy the professional who performs cervical cannulation is a gynaecologist who is acquainted with the procedure. On the other hand, we ruled out the use of an anti-reflux balloon in HyFoSy, because the patients studied are mostly nulliparous and cases of contrast media reflux are exceptional (0.9%, 1/107); therefore, higher pain on the VAS scale in the HSG group could be associated with the filling of the anti-reflux balloon in the cervix or uterine cavity, which has been associated with more pain and vagal reactions [43]. Finally, differences in the VAS score could be explained by a higher contrast volume instilled in HSG patients (8.8 ± 4.1 ml/pat vs. 4.5 ± 2 ml/pat in HyFoSy, *p* < 0.001), as a lesser distension of the uterine cavity is associated with lower perceived pain [43]; for this reason, we recommend using the lowest volume possible for fallopian tubes and uterine cavity assessment.

Due to the various limitations of the findings herein, overinterpretation of the results should be avoided. First of all, the techniques were performed in different centres by varying types of professionals and in routine clinical practice (real world-setting), implying differences that may influence the results, such as differences in contrast volume (the greater the volume, the greater the pain and the lower the rate of tubal obstruction in the HSG group) and the use of an anti-reflux balloon in the HSG group (greater pain). Nevertheless, both centres belong to the same Public Health Service and serve similar populations that are part of the whole geographical area of Madrid, and the procedures used are similar to those employed in other centres. Secondly, even if our statistical power were adequate enough to detect pain and anxiety differences, we have a relatively small patient cohort.

The main strength of our study is to provide data on the tolerability of HyFoSy in terms of anxiety, since there is scarce information about this in previous studies. It also supports prior data about less reported pain than with HSG. Another strength is its external validity; both study groups are similar (Table 1) and comparable to the population seen in public fertility centres in our country, as Spanish Law has very strict criteria for accessing this procedure (women aged between 18 and 40 years, without a previous healthy child and without pathologies that contraindicate fertility treatments or pregnancy) [33]. Moreover, one of the main values of real-world studies is that they are more generalisable than randomised clinical trials [28].

Notwithstanding, further research with a cost effectiveness analysis may be needed to better define the most appropriate way to improve care for patients undergoing tubal patency tests, either by endorsing changes in the protocol for HSG or by implementing HyCoSy/HyFoSy as a first-choice technique, as recommended by numerous authors [3, 7, 10, 11, 14, 15, 17, 18, 24–27], due to its additional advantages (ionising radiation and iodinated contrast are not employed, a comprehensive assessment of the uterus and the junctional area is possible in one single procedure, etc.). In this regard, van Rijswijk et al. are conducting the FOAM study: a randomised controlled trial with the aim of comparing the effectiveness and costs of management guided by HyFoSy or by HSG [44]. The primary outcome of the study is ongoing pregnancy leading to live birth within 12 months after randomisation, an interesting point that will allow us to know whether HyCoSy/HyFoSy is comparable to HSG in this respect.

## Conclusions

Our results suggest that HyFoSy is more tolerable than the current reference test (HSG), in terms of both pain and anxiety. This data could hypothetically support the possibility of changing the first-line diagnosis tool for tubal patency assessment, replacing HSG.

## Data Availability

The datasets used and/or analysed during the current study are available from the corresponding author on reasonable request.

## Abbreviations

HyCoSy: Hysterosalpingo-contrast sonography
HyFoSy: Hysterosalpingo-foam sonography
HSG: Hysterosalpingography
STAI: State Trait Anxiety Inventory
VAS: Visual Analogue Scale
ml: Millilitre
OR: Odds ratio
